# Supplementary material for: A Novel Graphene Oxide-Based Aptasensor for Amplified Fluorescent Detection of Aflatoxin M1 in Milk Powder
Source: Sensors (Basel). 2019 Sep 5;19(18):3840. doi: 10.3390/s19183840 (PMC6766899; doi:10.3390/s19183840)
Supplement: Supplementary file 1 [file sensors-19-03840-s001.pdf]

sensors

Article

A Novel Graphene Oxide-Based Aptasensor for Amplified Fluorescent Detection of Aflatoxin M_1 in Milk Powder

Xiaodong Guo [1,2,3,†], Fang Wen [1,3,†], Qinqin Qiao [1,3], Nan Zheng [1,3,4], Matthew Saive [2], Marie-Laure Fauconnier [2] and Jiaqi Wang [1,3,4,*]

[1] Key Laboratory of Quality & Safety Control for Milk and Dairy Products of Ministry of Agriculture and Rural Affairs, Institute of Animal Sciences, Chinese Academy of Agricultural Sciences, 100193 Beijing, China
[2] Chimie générale et organique, Gembloux Agro-Bio Tech, Université de Liège, Passage des Déportés, 25030 Gembloux, Belgium
[3] Laboratory of Quality and Safety Risk Assessment for Dairy Products of Ministry of Agriculture and Rural Affairs, Institute of Animal Sciences, Chinese Academy of Agricultural Sciences, 100193 Beijing, China
[4] State Key Laboratory of Animal Nutrition, Institute of Animal Science, Chinese Academy of Agricultural Sciences, 100193 Beijing, China
† The authors contributed equally.
* Correspondence: wangjiaqi@caas.cn; Tel: +86-10-62816069

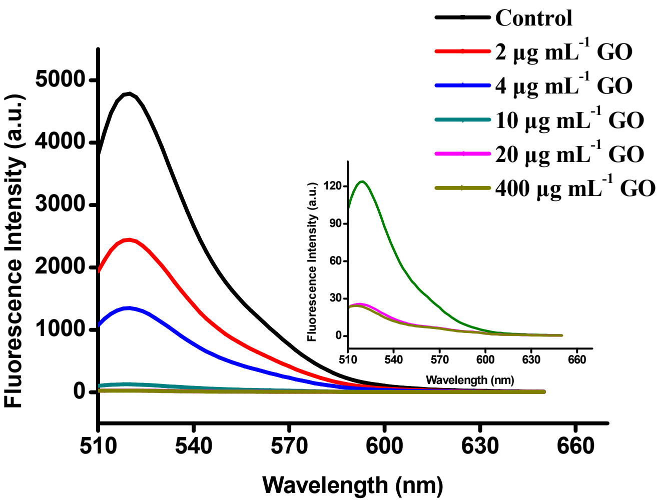

Figure 1. Fluorescence emission spectra of the aptasensor in the addition of GO at various concentrations. The experiment conditions are as following: λ_{ex} = 480 nm, 200 nM AFM_1 aptamer.

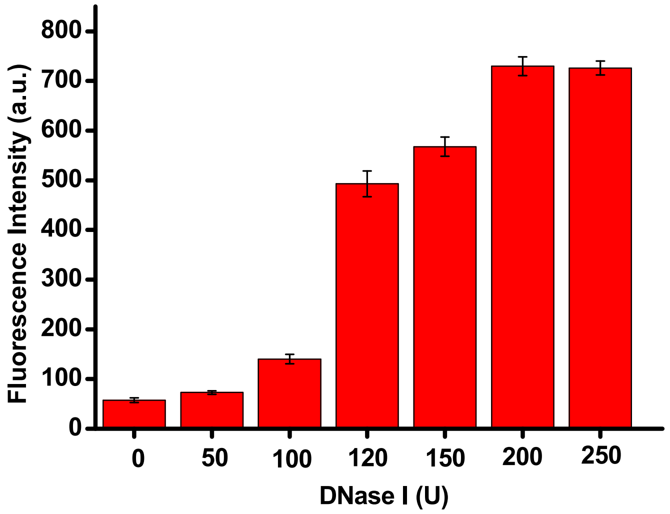

Figure S2. Fluorescence intensity with the addition of DNase I at various concentrations. The experiment conditions are as follows: Excitation and emission wavelength are at $\lambda_{ex}/\lambda_{em}$ = 480/520 nm, 200 nM AFM_1 aptamer, 20 μg mL^{-1} GO, 10 ng mL^{-1} AFM_1.
